# Supplementary material for: The direct and indirect costs of cardiovascular diseases in Brazil
Source: PLoS One. 2022 Dec 22;17(12):e0278891. doi: 10.1371/journal.pone.0278891 (PMC9778932; doi:10.1371/journal.pone.0278891)
Supplement: Attachment — Submitted filename: Response to reviewers - 2.docx [file pone.0278891.s002.docx]

9^th^ Nov 2022

**REBUTTAL LETTER**

**PONE-D-21-41019: The direct and indirect costs of cardiovascular diseases in Brazil**

Dear Pankaj Bahuguna, Ph.D.

We would like to thank you and the reviewers for your time to provide feedback on our paper. We have addressed all Journal requirements and the reviewers’ comments below.

**Journal Requirements**

**Comments to the Author:**

1. “Please review your reference list to ensure that it is complete and correct. If you have cited papers that have been retracted, please include the rationale for doing so in the manuscript text, or remove these references and replace them with relevant current references. Any changes to the reference list should be mentioned in the rebuttal letter that accompanies your revised manuscript. If you need to cite a retracted article, indicate the article’s retracted status in the References list and also include a citation and full reference for the retraction notice.”

**Response:** Thank you for raising this point. We extensively reviewed the reference list and, in this manuscript, we haven’t cited papers retracted.

**Reviewer #1**

**General comments to the Author:**

“Thank you for revising the manuscript and improving clarity for better understanding of the readers. However, the paper still needs language editing at few places and use of shorter sentences.”

**Response:** Thank you for your comments and for the opportunity to further improve our manuscript with your suggestions.

**Specific comments to the Author:**

**Abstract**

Line 24-25: It could be useful to know whether the cost mentioned here was overall cost/health system cost/societal cost and specifically for treatment?

**Response:** Thank you for your suggestion. We added the information that the costs were from health system and related to hospitalizations and outpatient care.

In **Lines 26-31**, now it says:

*“The calculation of direct costs for cardiovascular diseases (CVD) was made from the accounting sum of costs with hospitalizations and outpatient care found in the National Health System (Hospital Information System and Outpatient Information System), from 2017 to 2019, including the costs of treatment, such as medical consultations, medical procedures, and drugs. Regarding the indirect costs, they were measured by the loss of human capital, given the premature death, resulting in loss of productivity.”*

Line 27-28: Were these direct medical costs only? Please specify if so.

**Response:** Thank you for pointing out this omission. We added the costs’ information.

In **Lines 29-30**, now it says:

*“…including the costs of treatment, such as medical consultations, medical procedures, and drugs.”*

Line 29-30 can be rephrased to shorter sentences for better clarity.

**Response:** Thank you. We rephrased the sentence.

In **Lines 30-31**, now it says:

*“Regarding the indirect costs, they were measured by the loss of human capital, given the premature death, resulting in loss of productivity.”*

Results: Line 33: Higher burdens of what attributable to?

**Response:** Thank you. We now rephrased the sentence.

In **Line 33**, now it says:

*“Higher burden of CVD attributable to the consumption of salt,…”*

Conclusion Line 39: “Studies of this nature….” Is very broad scope and could be rephrased to narrower scope for clarity. Also authors may use shorter sentences in conclusion and should restrict to findings from the study than generic statements.

**Response:** Thank you. We now used shorter sentences restricted to our findings.

In **Lines 40-42**, now it says:

*“The attributable fractions to consumption of salt are the ones that cause the most effects on CVD, followed by saturated fat and trans fats, with direct and indirect costs being higher for males.”*

**Introduction**

Line 44: This line may be rephrased to “cardiovascular diseases (CVD) are one of the key challenges in health globally, since……………”

**Response:** Thank you for your suggestion. We rephrased the text using your suggestion.

Line 78: Please cite supporting references besides “few studies…….” Or may rephrase.

**Response:** Thank you for raising this point. Now we added two supporting references.

In **Lines 78-79**, now it says:

*“Few studies have been carried out with the objective of attributing the effect of nutrient consumption to the cause of CVD and its consequent costs [23,24].”*

Line 85-86 looks repetitive in lines 94-96 and may be better placed there.

**Response:** Thank you for spotting this. Now we deleted the lines 85-86 and placed only in **Lines 100-102**.

Lines 87-89 seems better suited to methodology section.

**Response:** Thank you for spotting this. Now we deleted **Lines 87-89** from the Introduction and added to methods section (**Lines 193-199**).

Line 99 could be rephrased to “Several databases were referred to collect and analyse CVD cost related information between 2017 and 2019 for Brazil.”

**Response:** Thank you for your suggestion. We rephrased the text using yours (now in **Lines 105-106**).

Line 114: There seems to be incomplete word in here. Whether its three sources or three databases, it is currently unclear to understand “three bases”.

**Response:** Thank you for spotting this. We made it clearer in the text that we used three databases to obtain the information needed to calculate indirect costs.

In **Line 121**, now it says:

*“Regarding indirect costs, three databases were necessary…”*

**Discussion**

“Line 293: The sentence could be rephased to “Findings from or study showed………….”

**Response:** Thank you for your suggestion. We rephrased the text using your suggestion.

**Reviewer #2**

**Comments to the Author:**

“Thank you for considering suggestions and revising the manuscript. The manuscript content is structured better and reads more logically now. The authors have addressed grammatical mistakes to a large extent. Policy relevance of this work is highlighted better than the previous version. Following are some of my suggestions/clarifications for the revised manuscript (Line numbers refer to the latest unmarked version):”

**Response:** we are very grateful for all the considerations. They were very valuable to improve our manuscript.

“Consider further grammatical checks: For example, replace burdens with burden (multiple places, replace servings with serving – Line 158, typos on Line 214-215, Replace manuscript with ‘study’ – Line 293, typo error on Line 295, typo on Line 375, consider replacing ‘inadequate diet’ with maybe unhealthy diet if you thing that is more reflective on Line 305. You may consider further similar grammatical checks in the manuscript.”

**Response:** Thank you for raising these points. We checked the whole manuscript looking for grammatical errors. Now the corrections are in red words.

“The revised introduction section makes a better read. However, on line 87, you go on to directly explain methods of indirect cost analysis. I would suggest you consider moving this to the methods section. The introduction section could give a brief overview of what all analysis (PAF, direct cost, indirect costs) is being aimed. Similarly, I found it difficult to establish relevance of Line 69-70 in context to the previous statement. What do you mean by recurrent costs here? My understanding is recurrent costs would be those attributed to hospitalisations and consultations (22% of costs)”

**Response:** Thank you for your comments. We moved the Line 87 and correlated aspects to methods section, and we added three paragraphs **(Lines 85-99)** with a brief overview of all analysis, as suggested. In **Lines 69-70**, we are talking about all kind of costs mentioned in previous statement (mortality, hospitalization and years of life lost). To make this information clearer, we have amended the text using “these recurrent costs”.

In **Lines 85-99**, now it says:

*“The Population Attributable Fraction (PAF) is a measure of public health impact that has been widely used by the World Health Organization, based on Global Burden Disease (GBD) data, in order to determine goals, prioritize interventions and build public policies [25,26]. In addition, the PAF has also been used to provide information on economic costs attributable to some risk factors, such as salt consumption [27].*

*There are two ways to estimate health costs for a disease. The first is “top-down”, going from the total values at the national level of the set of all diseases and, through a disaggregation process, arriving at the level at which the cost of the disease under analysis is found. The second is “bottom-up”, and through this method, estimates are made for a sample of cases and are extrapolated to the total number of individuals [28].*

*In Brazil’s context, it is possible to obtain the total direct costs related to a given pathology in the National Health System, which can be disaggregated by level of health care (outpatient and hospital), sex and age groups. Thus, the best approach to be used in Brazil is the top-down approach, from the perspective of public health services, based on health cost data, available in the information systems of the Ministry of Health [28].”*

In **Lines 69-70**, now it says:

*“Therefore, the growth of these recurrent costs represents an important problem for health systems, as well as for their socioeconomic impacts.”*

“For lines 137 to 143 – Can you clarify if adjusted relative risk values should be used for PAF calculation instead of relative risk?”

**Response:** Thank you for raising this point. To calculate the relative risk (RR) used in the PAF formula, it was calculated according to the distribution of our own variables. For this, we used a relative risk already established in the literature, from review studies or larger studies, such as the Global Burden Disease (GBD). These relative risks are adjusted for, at least, sex and age. Implicit in the way we characterize the RR function are some of the fundamental assumptions we make about relative risk. That is, the relative risk increases exponentially as the distance from the theoretical minimum level of risk exposure (y) increases, that there is no risk associated with exposure beyond the theoretical minimum level of risk exposure, and that both x and the level theoretical minimum risk exposure for an individual at exposure level x are the qth quantile of their respective distributions (the observed exposure distribution and the TMREL, respectively).

**Reference:**

Micha R, Peñalvo JL, Cudhea F, Imamura F, Rehm CD, Mozaffarian D. Association Between Dietary Factors and Mortality From Heart Disease, Stroke, and Type 2 Diabetes in the United States. JAMA. 2017 Mar 7;317(9):912-924.

“For lines 198 to 201 – Can you give any rationale/reasoning/references for choosing the six explanatory variables that have been chosen?”

**Response:** Thank you for your comments. The Mincerian equations from the study by Mincer (1974) show that the coefficients of age (proxy for experience), education and sex are significantly relevant to estimate individual income. For Brazil, due to regional/local and color/race disparities, the location variables "individual residence" and "color/race" are also of great importance. We added the reference in the text.

In **Lines 212-216**, now it says:

*“In this study, irreversible productivity losses due to the premature death of individuals aged 25 to 65 years caused by CVDs were considered. For this task, six explanatory variables for income that are available in the NHSS, and SIM databases were considered: state of the country in which the individual resides (residence), age, sex, education level, color/race, and marital status [39].”*

“Based on your results, specifically tables 1 and 2, can anything be commented/discussed on relationship between PAF and direct costs that you have observed?”

**Response:** Thank you for your advice. We added a paragraph in the discussion.

In **Lines 323-328**, now it says:

*“Although the highest CVD costs attributable to the consumption of salt, saturated fat and trans fats is among the 45-74 age groups, the consumption of these nutrients is inversely proportional to the age. One of the explanations for our findings is that, due to the greater number of CVD cases occurring in these age groups, the risk attributable to the consumption of these nutrients is added to the risk related to the age group, suggesting that preventive measures at younger ages can benefit the entire population in their future.”*

“In discussion, can you compare findings of high costs and mortality for males as compared to females with any existing evidence from Brazil to possibly validate your result?”

**Response:** Thank you for spotting this. In discussion, a study that addressed this issue with Brazilian data had already been mentioned. However, to reinforce our findings, we add another recent evidence.

In **Lines 337-345**, now it says:

*“Nilson et al. (2021) [24] estimated the economic effects and impact on health between 2013 and 2032 of the implementation of sodium reduction in processed foods in Brazil. They also reported that during this period, around 110,000 CVD male cases and 70,000 CVD female cases will be prevented. This estimate will result in a total savings of around US $ 220 million in medical costs for the Brazilian National Health System for the treatment of CHD and stroke. Moreover, corroborating our findings, a recent study carried out in Brazil showed that the costs attributable to excessive salt consumption were higher for males when compared to females, corresponding to 62% of the costs associated with hospitalizations and 53% of outpatient’s costs for CVD, attributable to salt consumption [27].”*

“For Table 4 from Line 290 onwards – I do not understand the unit used for your findings. The reported YLL is for what unit of observation? Is it for the entire cohort, per number of cases or what exactly?”

**Response:** Thank you for your question. The Years of Life Lost (YLL) for a cause are essentially calculated as the number of cause-specific deaths multiplied by a loss function, specifying the years lost for deaths as a function of the age at which death occurs. Table 4 shows the total years of life lost attributable to the consumption of nutrients (salt, saturated fat and trans fats) by age group and sex. The unit of measurement is actually "years", as presented in the Global Burden Disease (GBD) and as presented in other publications (please, see the doi <https://doi.org/10.1371/journal.pone.0235514.t003>, as example).

“In discussion section, can you compare your indirect cost findings to any other relevant literature?”

**Response:** Thank you for your suggestion. The problem with making comparisons in relation to indirect costs lies precisely in the difference between the methodologies adopted in studies involving this theme. Recent work by Nilson et al (2020), assessed the years of life lost, however, it did not calculate indirect costs. Rasmussen's study (2015), for example, which carried out an analysis of indirect costs, addressed other methodological aspects, such as absenteeism, presenteeism and early retirement. So, we decided to add a few words to the discussion referring to a limitation of comparing these data given these previous explanations.

In **Lines 454-455**, now it says:

*“Besides, the fact that other studies use different approaches to calculate the indirect costs makes it difficult to compare the results [12,64,65].”*

**Patrícia Moreira**

On Behalf of co-authors
